# Stakeholders’ perspectives on integrating the management of depression into routine HIV care in Uganda: qualitative findings from a feasibility study

**DOI:** 10.1186/s13033-021-00486-8

**Published:** 2021-07-01

**Authors:** Rwamahe Rutakumwa, Joshua Ssebunnya, James Mugisha, Richard Steven Mpango, Christine Tusiime, Leticia Kyohangirwe, Geoffrey Taasi, Hafsa Sentongo, Pontiano Kaleebu, Vikram Patel, Eugene Kinyanda

**Affiliations:** 1 MRC/UVRI & LSHTM Uganda Research Unit & Senior Wellcome Trust Fellowship, Mental Health Section, 50-59 Nakiwogo Street, Entebbe, Uganda; 2 Butabika National Referral Mental Hospital, Old Port Bell Road, Kampala, Uganda; 3 Kyambogo University, Kampala, Uganda; 4 STD/AIDS Control Program, Ministry of Health, Ministry of Health, Kampala, Uganda; 5 Mental Health Division, Ministry of Health, Kampala, Uganda; 6 Director of the MRC/UVRI & LSHTM Uganda Research Unit, Kampala, Uganda; 7 Department of Global Health and Social Medicine, Harvard Medical School, Boston, MA USA; 8 Department of Psychiatry, Makerere University, Kampala, Uganda

**Keywords:** Depression, HIV, ART, Mental health, Uganda

## Abstract

**Background:**

HIV/AIDS continues to be a major global public health problem with Eastern and Southern Africa being the regions most affected. With increased access to effective antiretroviral therapy, HIV has become a chronic and manageable disease, bringing to the fore issues of quality of life including mental wellbeing. Despite this, the majority of HIV care providers in sub-Saharan Africa, including Uganda’s Ministry of Health, do not routinely provide mental health care including depression management. The purpose of this paper is to explore stakeholders’ perspectives on the feasibility and acceptability of integrating depression management into routine adult HIV care. The paper addresses a specific objective of the formative phase of the HIV + D study aimed at developing and evaluating a model for integrating depression management into routine HIV care in Uganda.

**Methods:**

This was a qualitative study. Data were collected through in-depth interviews with 11 patients at enrollment and follow-up in the pilot phase, and exit interviews with 11 adherent patients (those who completed their psychotherapy sessions) and six non-adherent patients (those missing at least two sessions) at the end of the pilot phase. Key informant interviews were held with four clinicians, five supervisors and one mental health specialist, as were three focus group discussions with lay health workers. These were purposively sampled at four public health facilities in Mpigi District. Data were analysed thematically.

**Results:**

Patients highlighted the benefits of treating depression in the context of HIV care, including improved adherence to antiretroviral therapy, overcoming sleeplessness and suicidal ideation, and regaining a sense of self-efficacy. Although clinicians and other stakeholders reported benefits of treating depression, they cited challenges in managing depression with HIV care, which were organisational (increased workload) and patient related (extended waiting time and perceptions of preferential treatment). Stakeholders generally shared perspectives on how best to integrate, including recommendations for organisational level interventions–training, harmonisation in scheduling appointments and structural changes–and patient level interventions to enhance knowledge about depression.

**Conclusions:**

Integrating depression management into routine HIV care in Uganda is acceptable among key stakeholders, but the technical and operational feasibility of integration would require changes both at the organisational and patient levels.

## Background

HIV/AIDS continues to be a major global public health problem, having claimed over 33 million lives so far, and with an estimated 38 million people living with the disease globally [1]. The Eastern and Southern Africa regions are the regions most affected by HIV, being home to more than a half (54%) of the total number of people living with HIV in the world and with a growing number of new infections [1].

With increased access to effective anti-retroviral therapy (ART), HIV is gradually becoming a chronic and manageable disease with prolonged survival bringing issues of quality of life including mental wellbeing into the forefront [2–4]. Indeed, HIV/AIDS has been associated with a number of mental health problems that include depression, anxiety disorder, alcohol use disorder, suicidality and neurocognitive disorders [5, 6]. In previous studies undertaken in the study area by this research group, rates of depression among HIV patients of 8 percent in urban areas [5, 7], and 19.5 percent in rural areas [7] were observed. In both these studies the rates were higher in females than in males, at 15.6 percent versus 8.2 percent [7], and at 9.6 percent versus 4.1 percent [5]. Depression in HIV/AIDS not only leads to severe psychological distress, it has been associated with a number of negative clinical and behavioural outcomes [8].

Despite the importance of mental health problems, particularly depression, the majority of HIV care services in sub-Saharan Africa lack mental health care including services for treating depression [9]. Depression among people living with HIV and AIDS (PLWHA) continues to be a threat to the success of ART, hindering adherence and subsequently negatively affecting clinical outcomes [10, 11]. There is growing evidence of the high prevalence of depression and its damaging effects among PLWHA, and that this is partly attributable to absence of depression treatment in HIV care programs, severe shortage of mental health professionals and understaffing of the HIV clinics [5, 9, 12]. Indeed, in sub-Saharan Africa up to 30 percent of depressive disorders have been associated with HIV/AIDS [8]. Depression among PLWHA not only affects quality of life [3], but has been associated with a number of other negative behavioural and clinical outcomes such as more rapid HIV disease progression and mortality [13–15], poor adherence to HIV treatment, risky sexual behaviour and increased utilization of health facilities [8, 16, 17]. Indeed, global predictions indicate that both HIV/AIDS and depression will be the first two leading causes of disability globally by 2030 [18, 19]. Despite this, the majority of HIV care providers in sub-Saharan Africa do not routinely provide mental health services including depression management [12, 20].

In a recent meta-analysis, a 31 percent pooled depression prevalence was found among PLWHA in Uganda, almost 10 times higher than the estimated prevalence in the general population [21]. With an estimated 1.4 million people living with HIV/AIDS in Uganda, this poses a major challenge in HIV care despite the success in the scale up of ART and consequently increasing mortality [8, 22]. In response to this absence of mental health care in HIV programs, the Uganda National HIV and AIDS Strategic Plan (2015–2020) has called for the integration of mental health and other chronic conditions in HIV care so as to further improve the quality of care and treatment [22]. To operationalise this policy recommendation, the Ministry of Health released guidelines for the treatment of HIV calling for the assessment and management of depression as an integral part of HIV care programs [23].

Although the need for integrating HIV and mental health services is indisputable, challenges exist in implementing a service integration model that is effective against mental disorders and is cost-effective to the health system [24]. Such a model should also be acceptable and feasible for improving both mental health and HIV treatment outcomes. It is thus imperative that the model is informed by the perceptions and experiences of relevant stakeholders. There is limited data on the integration of depression management in HIV/AIDS in sub-Saharan Africa. To date we are aware of only two studies that have been undertaken, the first being the INDEPTH-Uganda Trial that was implemented in Uganda and evaluated two task-shifting approaches to depression care–a protocolised model versus a model that relies on the clinical acumen of trained providers to provide depression care [9]. The second study was the COBALT Trial, which was undertaken in South Africa, and evaluated a nurse led depression integration model in HIV care [25]. While the COBALT trial has not published its results, the INDEPTH-Uganda trial found that protocolised delivery of therapy was superior to merely providing treatment guidelines.

In support of the Ministry of Health policy recommendation, the Mental Health Section of the Medical Research Council/Uganda Virus Research Institute & London School of Hygiene and Tropical Medicine Uganda Research Unit in partnership with the STD/AIDS Control Program of the Ministry of Health is implementing a 5-year study named HIV + D Trial. The HIV + D Trial aims to develop and evaluate at trial a model for integrating the management of depression into routine HIV care in Uganda [26]. This proposed HIV + D Trial will evaluate the effectiveness and cost-effectiveness of a stepped care depression integration model that will be delivered by the HIV care team at Public Health Care Facilities (PHCFs) in partnership with lay health workers who will be derived from either members of the village health team (VHT; the first level of the Ugandan health care system) or lay health workers (expert clients living with HIV). The intervention will be coordinated by the existing HIV counsellors (general nurses who have received training in HIV counselling, working at PHCFs), and will be supported by mental health professionals (psychiatric clinical officers or psychiatric nurses) based at Health Centre IVs (health sub-districts serving as referral centres for lower health facilities), district and regional referral hospitals. This paper is based on data from the formative phase of the HIV + D intervention that has already been implemented, whose principal aim was to develop an intervention model for the HIV + D trial. In this paper, we explore stakeholders’ perspectives on the feasibility and acceptability of integrating the management of depression into routine adult HIV care in Uganda.

## Theoretical framework

Our choice of theoretical model of integrated care derives from what has been referred to as realist models. Although we did not identify previous similar studies that have used the realist model, we consider the model suitable for guiding the research. Realist models generally highlight the significance of context in developing integrated care programmes that achieve desired outcomes [27]. With a grounding in empirical evidence these models seek to lay out realistic pathways to successful integration by, for example, identifying the contextual variables (such as strong leadership and organisational culture) that would be key for successful integration in a specific programme area (for instance institution of multidisciplinary teams) [28].

Two main considerations influenced this choice. First, realist models provide for contextual specificity. This enabled us to draw on our observations during the formative study (on which this paper is based) undertaken at four public health facilities, from which practical lessons were drawn in order to verify what aspects of integration were considered acceptable/unacceptable. Second, integration within realist frameworks is targeted at specific programmes/services (in our case, integrating the management of depression into routine HIV care).

Accordingly, our concept of integration for the purpose of this paper is the one that engenders establishment of multi-purpose health service delivery points in one location, providing different clinical and related services to a catchment population [29]–in this case, HIV patients who may need depression-related care in addition to their regular HIV-related services. Such integration therefore is essentially aimed at addressing disjointedness and promoting coordinated care in the provision of services so that the user can easily navigate the varied services that they need on a single trip to the facility [29].

We also find Benzer and colleagues’ [30] theoretical perspectives on factors influencing success of integrated care pertinent to our purpose. In their grounded theory on integration of mental health services into primary care the authors identify, among others, organizational factors (including resources, training and work design), provider experiences (such as time pressures arising from redistribution of workloads) and patient factors (conceptualized as the surrounding environment) as key in shaping success of integration.

In this paper we will draw on these theoretical perspectives to interpret and discuss our findings.

## Methods

### Design

This was a qualitative study, part of the broader formative phase of the HIV + D intervention whose general objective was to develop and evaluate a model for the integration of depression management into routine HIV care in Uganda. The formative phase was guided by three specific objectives: i) to adapt the MANAS intervention–a stepped-care collaborative model for the integration of mental health care into primary health care that was successfully evaluated in India–to the Ugandan HIV care context; ii) to adapt the Healthy Activity Program (HAP) [31], a form of Behavioural Activation Therapy. The HAP is a brief psychological treatment for depression that was successfully evaluated in India–to the Ugandan HIV care context; and iii) to evaluate the acceptability and feasibility of an intervention that will integrate the management of depression into routine HIV care in Uganda (the “HIV + D” intervention). By exploring stakeholders’ perspectives on the feasibility and acceptability of integrating the management of depression into routine adult HIV care in Uganda, this paper directly addresses Specific Objective III of this formative phase.

### The HIV + D intervention

**Fig. 1:**
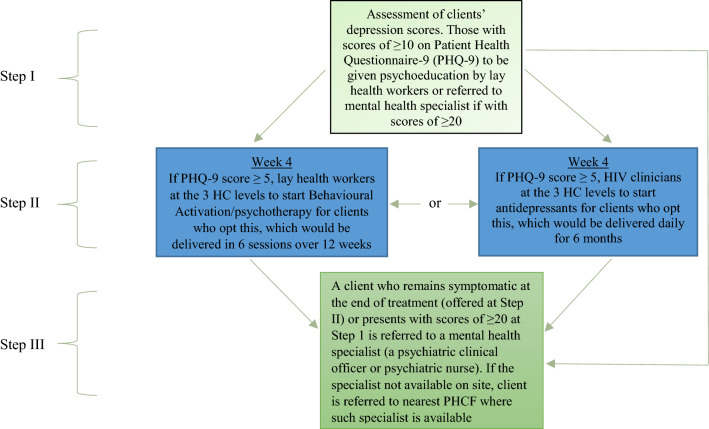
The stepped care (HIV+D) intervention

The HIV + D intervention was a stepped care collaborative model that was initially conceived to be delivered in three steps, and uniformly across the three levels of health care (III, IV and district hospital) where the intervention was implemented. At all these levels the focal point was the HIV clinic. The intervention is summarised in Fig. 1 below.

### Study setting

This formative study was undertaken at four public health care facilities (PHCFs) in Mpigi District in central Uganda, a district that has had a long term relationship with MRC/UVRI and LSHTM Uganda Research Unit. The four selected PHCFs represented three levels of the Ugandan health care system: Butoolo and Buwama, with level three health centres (HCIII); Mpigi, with level four health centres; and Nkozi, with a hospital level health facility. Mpigi district is a semi-urban, densely populated district with both urban and rural populations. The district comprises 19 PHCFs which offer varying levels of care, graded as HCI, II, III, IV and a hospital, with the latter three HC levels (where the intervention was delivered) offering the same services. The HCIV (Health sub-district) serves as the referral point for the lower HCs. The health facilities run HIV clinics weekly. These clinics are staffed with general health workers, counsellors and lay health workers (expert clients who live with HIV and support other PLWHA). At the lowest level (HCI) within each village, volunteers from within the community are nominated by community members to form Village Health Teams (VHTs) entrusted with taking care of health matters of villages where they live. There is no built structure or qualified health staff at this level.

It was important to get a perspective from each of these levels of care for two reasons. First, these different levels represent the range of HIV care services in the country. Second, the perspectives from the different levels were to inform the proposed HIV + D trial that was to be undertaken in 40 PHCFs represented by these levels of care.

### Study participants

Multiple stakeholders including mental health specialists, healthcare workers (clinicians), and service users (including PLWHA) were purposively sampled for inclusion in development of the HIV + D intervention. These were involved at the different levels (HCIII, IV and the district hospital) of the health care system where the study was undertaken. For the depressed PLWHA, eligibility for inclusion in the study was (i) an HIV positive status with evidence of a certificate, (ii) registration with an HIV clinic in Mpigi district, (iii) being 18 years and above, (iv) ability to communicate in either English or Luganda (the local language) and (v) a score on the Patient Health Questionnaire-9 (PHQ-9) [32] of 10 and above. For the other stakeholders, those eligible for inclusion were the ones participating in the delivery of the HIV + D intervention.

In pursuit of Specific Objective III of the formative phase (to evaluate the acceptability and feasibility of an intervention to integrate the management of depression into routine HIV care in Uganda) which we directly address in this paper, data were collected from three categories of participants: (i) the Healthy Activity Program/Behavioural Activation (BA) core team that delivered BA psychotherapy–these were the lay health workers who were also living with HIV and supervised by an HIV counsellor or nurse; this core team was supported by mental health specialists, (ii) clinicians who delivered antidepressants and (iii) study participants (depressed PLWHA) who were attending ART clinic (at the four selected public health care facilities) with moderate/severe symptoms of depression (those scoring 10–19 on the Patient Health Questionnaire-9 [PHQ-9], which was validated in Uganda) and had been purposively selected to participate in the HIV + D intervention. Information on sample sizes for the different categories of participants is summarised on Table 1 below.

**Table 1:** Summary of participant recruitment and data collection exercises

Participants	Number enrolled	Source of data	Baseline (month 0)	Midline (3rd month)	Endline (6th month)	Exit interview (6th month)
HIV patients (PLWHA)	131 (100 females; 31 males)	Serial in-depth interviews with 6 HIV patients on psychotherapy and 5 on antidepressants	11	11	8	
		Exit in-depth interviews with 11 adherent (those who completed all the psychotherapy [n = 9] and antidepressant [n-2] sessions) and 6 non-adherent (those who missed ≥ 2 sessions) HIV patients. These had not been interviewed				17
Lay health workers	8 (5 females; 3 males)	Serial focus group discussions (FGDs)	1 FGD	1 FGD	1 FGD	
Clinicians	4 males	Serial key informant interviews	4	4	4	
Supervisors	4 (3 males; 1 female	Serial key informant interviews	4	3	3	
Mental health specialists	4	Serial key informant interviews	0	0	1	

### Data collection procedures

Using semi-structured interview topic guides, in-depth interviews were conducted with PLWHA, key informant interviews were conducted with clinicians, supervisors and a mental health specialist, and focus group discussions were held with lay health workers (expert clients). Additionally, exit interviews were conducted with adherent and non-adherent HIV patients–it is worth noting that we registered non-adherence among the PLWHA receiving psychotherapy and none among those on antidepressants. These exit interview participants were a subset of PLWHA attending ART clinic but who had not been interviewed. More information on data collection processes and topics covered is highlighted on Tables 1 and 2 below.

**Table 2:** Topics covered during interviews/focus group discussions with participants

Participant category	Topics covered during interview/focus group discussion
PLWHA	**At baseline**: depression awareness, depression treatment experiences and feasibility of integration **At midline**: experiences with those delivering the treatment, challenges during treatment and recommendations for change **At endline**: changes in health since the last therapy session, challenges encountered, what worked/did not work well and recommendations for change
Adherent and non-adherent HIV patients (a subset of PLWHA who had not been interviewed) participating in exit interviews	Experiences and reasons for adherence/non-adherence
Lay health workers	**At baseline**: perspectives on depression and psychotherapy, the process of delivery of psychotherapy, and feasibility of integration **At midline**: experiences and challenges of delivering psychotherapy and recommendations for improving the treatment, ease of applying decision support algorithm **At endline**: experiences of managing depression and with patients, challenges of managing depression and perspectives on integration, ease of applying decision support algorithm
Clinicians and supervisors	**At baseline**: depression prevalence, patients’ care-seeking behaviour, experiences with depression patients, perspectives on antidepressants and its delivery, and integration **At midline**: experiences and challenges of delivering antidepressants, ease of applying decision support algorithm and recommendations for improvement in treatment delivery **At endline**: experiences of depression management, barriers of treatment delivery and recommendations, ease of applying decision support algorithm and perspectives on challenges and opportunities of integration
Mental health specialists	Experiences of depression management, barriers of treatment delivery and recommendations, and perspectives on challenges and opportunities of integration

All interviews and focus group discussions were conducted/moderated by research assistants who were psychiatric nurses, and were audio taped and transcribed verbatim. Interviews and focus group discussions with patients and lay health workers were conducted/moderated in the local language (Luganda). These were then transcribed in Luganda and translated to English by the interviewers/moderators who conducted the respective interview/focus group discussion. Interviews with clinicians and supervisors were conducted and transcribed in English. The interviews on overage lasted 45 to 60 min while focus group discussions lasted one to two hours.

### Data analysis

Two experienced qualitative researchers conducted the data analysis at different time periods. The first researcher conducted a data-driven analysis aimed at an initial in-depth exploration of the data. Building on the output from this analysis, the second researcher conducted an analysis driven by the specific research objective related to the feasibility and acceptability of integrating the management of depression into routine HIV care. The outputs of the analysis from the two researchers were reviewed by the study team and found to be generally consistent and accurate.

The data were analysed using thematic analysis techniques, which involve the identification, analysis, organisation, description, and reporting of themes found within a data set [33]. The data were coded with the help of a thematic framework initially developed from the emerging themes and refined based on concepts borrowed from our theoretical framework. Using MS Excel, the key themes and sub-themes in the thematic framework were classified on a matrix, which was then used to capture relevant pieces of data that illustrated the themes.

## Results

**Fig. 2:**
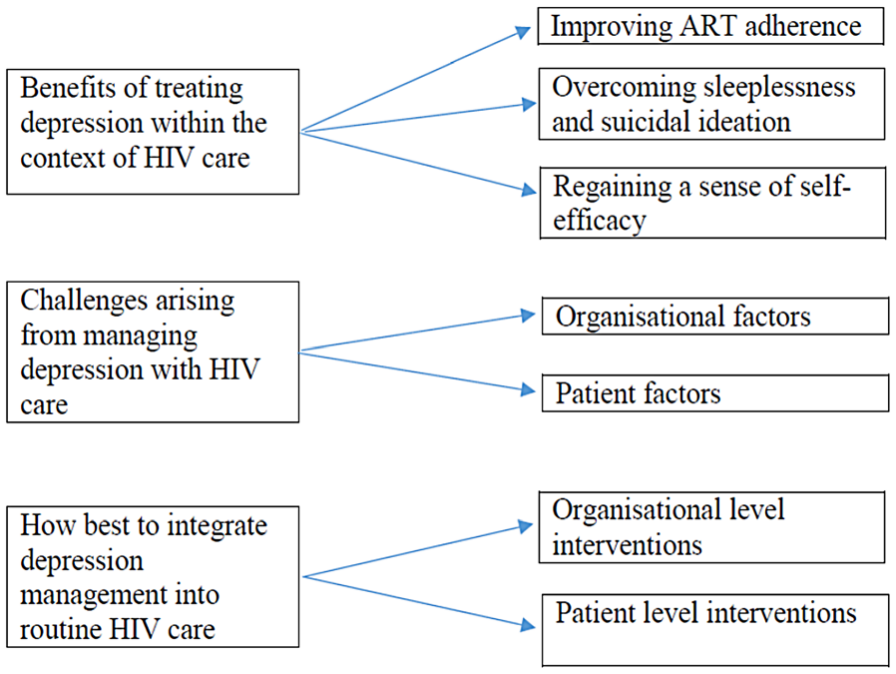
Conceptual figure highlighting the themes emerging from the data and their relationships

In the findings below we present perspectives of the cross section of stakeholders on the acceptability and feasibility of integrating the management of depression into routine HIV care in Uganda. It is worth noting that stakeholders’ perspectives on feasibility related to only two aspects: the technical aspect, in which we sought to assess whether the organisation has the necessary technical resources, including expertise, for successful implementation of the integrated model; and the operational aspect by which we examined how integration fits within the existing environment, with a focus on suitability of the public health care facilities’ physical infrastructure for implementing the integrated model. Also noteworthy is that while there was no noticeable variance in perspectives among the different stakeholder categories, concurrence was most evident among patients and clinicians who were the most outspoken with regard to their positive attitudes towards acceptability and feasibility of integration (Fig. 2).

### Benefits of treating depression within the context of HIV care

Irrespective of choice of treatment, participants generally had a positive view about the impact of depression treatment within the context of HIV care. This view was mainly expressed by PLWHA but was notably also echoed by other stakeholders including supervisors and lay health workers. It is against this backdrop that PLWHA persistently called for the harmonisation of HIV and depression services as expressed in the following two excerpts:

*“I think it would be good [to] see how best you can integrate depression treatment into our routine HIV care. I am sure that after this study, these medical forms will eventually be put into our ART files so that those health workers will be able to know that we are on treatment for depression. So the treatments can be harmonized such that we are given the same appointment dates for antidepressants and ART” (Nkozi, Antidepressants, Patient 1, Midline Interview)*.

*“I suggest that at every ART facility there should be enough health workers trained to handle depression … because it would be fair if every person coming for their ART refills can be assessed and treated for depression. It will also help patients who are failing to suppress because of depression” (Buwama, Psychotherapy 1, Endline Interview).*

We viewed these findings as a strong indicator of the acceptability of integrating depression treatment within HIV care.

We also note that the positive feedback from stakeholders as earlier noted was shared mostly at endline and often at midline, although there were a few cases in which patients shared the feedback at the baseline interview (immediately preceding the start of psychotherapy or antidepressant medication). In these cases, the patient had registered early benefits from the psychoeducation (as described earlier in the methods), an initial single session depression therapy that was offered to all participating patients as a first step towards the intervention. Patients consistently highlighted a number of physical and mental health benefits of depression treatment.

### Improving ART adherence

Perhaps the most pertinent benefit of depression treatment was that it potentially enhanced HIV treatment outcomes by improving ART adherence. With regard to improving adherence, our analysis reveals both direct and indirect pathways through which this occurred. One of the direct pathways was that depression treatment helped the despondent patient regain hope in their wellbeing.

*“[As a result of my treatment for depression] I have also been able to regain my hope. I no longer fall sick very often like how it used to be earlier on before being enrolled into this study”(Buwama, Psychotherapy 1, Endline Interview).*

This hope was usually accompanied by a renewed disposition on the part of participants to adhere to their ART regimen. Reflecting on her own depression treatment experience, the same participant credited the treatment for improved ART adherence, and ventured further to strongly recommend the treatment to anyone living with HIV as a way to avoid poor adherence to ART treatment which may lead to the clinician putting you on second line ART.

*“Depression treatment [counselling] is very helpful among people living with HIV/AIDS. If you have not been taking your medication well it helps you to take it and you end up feeling well…. So I would strongly recommend that all people living with HIV/AIDS should receive this [depression] treatment. If that is done we shall have few people on second line treatment regimen which is hard to get… yet the number of people on second line is increasing” (Buwama, Psychotherapy 1, Endline Interview).*

On the other hand, one of the indirect pathways through which depression treatment would lead to improved ART adherence, according to some patients, was by easing disclosure in discordant relationships. Non-disclosure was considered to be a significant barrier in ART adherence in a sense that treatment doses were sometimes administered late or even skipped if the environment was not discreet enough to facilitate administration of the dose without the partner’s awareness. For patients in this situation therefore, disclosure was an imperative for enhancing adherence and their own health. Yet for one of these patients, for example, the act of disclosing was complicated further by earlier indications from the partner that she would abandon him if they were found to be discordant and she is negative. This participant credited the psychotherapy associated with depression treatment for having equipped him with the skills and courage to disclose.

*“For my case the cause of depression was that I was in a discordant relationship in which I was HIV positive yet my wife was negative [and she did not know my status]. So that kept me worried about how I would break such disturbing news to her…. And also basing on the statements that she used to make that “in case we test… and I am found negative when he is positive, I will just divorce him”…. It is from this study that I acquired skills to disclose to her. Eventually I informed her about my status…. (Nkozi, Psychotherapy 2, Nkozi, Baseline Interview).*

Besides improving ART adherence, participants described other experiences in which psychotherapy enhanced HIV treatment outcomes. This was particularly so in instances where a patient was adhering to the ART regimen and still would not register viral suppression, but did show viral suppression after psychotherapy, in which case psychotherapy was considered as having enabled the body to respond better to ARVs.

*“They used to tell me that I was taking my ARVs badly; they even thought I was taking alcohol yet I was not. But after receiving psychotherapy, I was amazed when the clinicians told me that I was taking my medication well. That amazed me a lot because for seven years I had never suppressed, not until now. However, I realized that [before psychotherapy] I used to take my ARVs but would still worry a lot and even keep thinking about suicide – so that made me not to suppress the virus” (Buwama, Adherent 2, Exit Interview).*

### Overcoming sleeplessness and suicidal ideation

Another reported benefit of depression treatment was that it helped overcome sleeplessness. This apparently was the most frequently cited benefit. With the exception of one who experienced acute side effects from antidepressants and had to discontinue the therapy, participants from both the antidepressant and the psychotherapy groups were unanimous in their view that the treatment had markedly improved their sleep and general wellbeing.

*“Before I was initiated on that [depression] treatment, I would go to bed and keep awake –with no sleep and I would have intrusive thoughts of why I do not die and leave this world. I used not to have sleep at all. I would only sleep when it was approaching daytime, but when I started the treatment, I can now sleep soundly and the bad thoughts I used to have are no more” (Butoolo, Antidepressants, Midline Interview).*

This view was echoed by supervisors, as one noted:

*“Yeah because those on treatment… were not sleeping at first but after putting them on treatment they were now sleeping well. Some were not eating after treatment they started eating well and doing their work as usual” (Buwama Supervisor, Endline Interview).*

Perhaps as a knock-on effect of being able to sleep normally and to avoid too many thoughts, several participants also talked about having overcome suicidal ideation.

*“The [antidepressant] tablets are good…. I never used to sleep but now I can. The second benefit [of depression treatment] is that I used to have bad thoughts of taking many ART tablets [attempting suicide] so that I die since I was living alone. Fortunately, I no longer have them, and feeling lonely has reduced” (Mpigi, Antidepressants, Midline Interview).*

*“I used to lack sleep but right now I sleep well, I used to lack appetite but now I have a lot of appetite…. The tablets also helped me to overcome the many thoughts that I used to have such as suicidal ideations and thoughts on self-isolation” (Nkozi, Antidepressants, Patient 1, Endline Interview).*

### Regaining a sense of self-efficacy

The other notable benefit of depression treatment was that treatment recipients regained a sense of “self-efficacy,” that is, a belief in one’s own ability to influence events that affect their lives [34]. This was perhaps not surprising given the generally positive outlook that majority of participants projected in their feedback on the treatment. Participants observed that the treatment enabled them to regain control over their lives by empowering them with knowledge about a health condition (depression) they suffered from but did not understand, and by instilling in them a belief in their own agency in the efforts to resolve their health problems.

*“I used to think a lot and I would end up falling sick because of those many thoughts but nowadays that no longer happens, because I have applied some of the skills that I learnt during counselling [psychotherapy] such as, letting things go, avoiding overthinking, breathing in and out in order to relax…. However, I also believe that if I happen to experience any signs and symptoms I will be able to overcome them before they cause much harm to me. I now also understand depression illness in detail, I have learnt how to overcome it” (Buwama, Psychotherapy 1, Endline Interview).*

This sentiment was also highlighted during a group discussion with lay health workers, with one of the participants noting:

*“Counselling [psychotherapy] has helped patients a lot because… those who are being discharged act as ambassadors after they have learnt how to overcome their problems. Psychotherapy helps to empower someone with skills that help them to encounter any future challenges/problems” (Focus Group Discussion with Lay Health Workers, Midline).*

### Challenges arising from managing depression with HIV care

While the feedback from those receiving treatment for depression was overwhelmingly positive, our analysis of data revealed potential barriers to the integration of depression management into routine HIV care. Drawing on their own experiences from the study, clinicians from the four study sites were unanimous in their view that the integration presented some challenges that impacted both patients and clinicians. These challenges were mainly organisational factors, particularly increased workload for clinicians, and patient factors including extended waiting time at the health facility and patients’ perception of preferential treatment.

### Organisational factors

Clinicians cited a key organizational factor in the form of increased workload and associated time pressures as one of the key challenges to integrating depression management into routine HIV care. They acknowledged that the addition of depression care onto their already stretched schedule of HIV care would increase burnout, at least in the short term. This ordinarily would have engendered the clinicians’ disinclination towards the integrated service delivery model. However, what was also notably apparent in the clinicians’ narratives around increased workload was that they maintained a positive attitude towards integration of the two services, and expressed willingness to adopt the model. For some clinicians, adoption of the integrated model despite the increased workload was justified given that this would likely benefit them by making work less demanding in the longer term than under the current model. One clinician noted, for example, that the increased workload would be easier to manage than having patients whose HIV viral load was not suppressed, itself the function of untreated depression among the HIV patients.

*“Ah… work load! I do not want to say it because it is easier to manage. As for me the time I have managed this depression and HIV and now a third element of non-suppression [non suppression of HIV viral load], it is easier to manage a depressive patient, and it is very important to deal with a depressive patient than with one whose HIV viral load is not suppressed…. When someone’s HIV viral load is suppressed it saves time; we just give them drug refills. However for those whose HIV viral load is not suppressed we have to go into clinical management including investigating why their HIV viral loads are high. So when you manage depression it will be like a short cut for you to get a suppressed patient. So as for me, I do not see it as a work load, we just need to appreciate it and when we do it well we shall be saving the time which we would have spent on the non-suppressed…” (Butoolo Clinician, Midline Interview).*

Another clinician highlighted the magnitude of the increased workload but implied that this was acceptable since the workload was expected to reduce with time.

*“That is a challenge because sometimes you have over 100 clients in a day – you have to get time and also attend to others [and] counselling [psychotherapy] needs time …. You know it is quite challenging… like you have just started to come in, you feel that you have added some extra work. But as time goes on, you get used actually, you go on adjusting slowly by slowly in fact at the end of the day you find that you have not added any extra work. So the screening process becomes simple and easier, then the counselling process [psychotherapy] is also decreasing and you get used to it” (Mpigi Clinician, Endline Interview).*

### Patient factors

*Extended waiting time.* The extended time spent waiting at the clinician’s office was identified as one of the key patient related challenges of integrating depression into HIV care. Clinicians worried that longer waits than was the case before integration would in turn generate discontent among patients who might perceive this as a deterioration in the quality of care. This perception was particularly likely among those visiting for a simple procedure, such as an HIV viral load check, but are being made to wait much longer. One clinician observed:

*“So now it could be time for the [other] client to get the assessment but the time you spend assessing depressed clients you alter the time for the other client who just came probably for a viral load check and should be gone quickly. So someone who would have spent 15-20 minutes at the facility may end up waiting 30 minutes. So I think that will affect the routine flow and again that is waiting time mainly” (Buwama Clinician, Baseline Interview).*

On the same note, a lay health worker observed:

*“You actually need a lot of time to speak to a depressed client… some cry during the sessions and as such you have to give them time to do so…. Sometimes you would have like three patients waiting for you to see them, and as a result these other patients would end up waiting for long periods of time” (Focus Group Discussion with Lay Health Workers, Endline).*

*Patients’ perception of preferential treatment.* Another key patient related challenge of integration was the patients’ perception that clinicians were giving preferential treatment to other patients, which resulted in longer waits at the clinician’s office. For example, one clinician was concerned that some patients, especially those that have already been waiting for long, might feel ignored when they see their counterpart who is depressed and in need of urgent doctor’s attention being prioritised over them.

*“…that was a challenge–for example I have a queue there, clients have been waiting and this client comes in and of course she may need immediate attention which I was not able to give to them…. The other ones may see it as [giving others preferential treatment]” (Nkozi Clinician, Midline Interview).*

### How best to integrate depression management into routine HIV care

While stakeholders spoke highly about the benefits of integrating depression management into routine HIV care, they also identified some necessary conditions for the successful operationalisation of this service delivery model. We identified mainly two of these conditions, namely organisational (including training to enhance clinical expertise, harmonisation in scheduling appointments, and structural changes) and patient level interventions to enhance knowledge of depression. We viewed the possible fulfilment of these conditions, which might not require heavy resource investment, as indicative of the feasibility of integrating depression treatment into routing HIV care.

### Organisational level interventions

*Training to enhance clinical expertise.* At the organisational level, one of the key conditions for the successful operationalisation of the integrated service delivery model was training to enhance clinicians’ competence in delivering both HIV and, especially, depression care. This suggestion came particularly from clinicians, who had a background in HIV care and would be expected to manage depression among their HIV patients. The training reportedly would address the current knowledge/skills gap among clinicians, including an understanding of depression as a common comorbidity among HIV clients.

*“I think everything will be based on the staff and they should acknowledge the new program [integrated services], they should be able to know that really this [depression] happens, people with HIV can have depression concurrently, and then as I said we need continuous refresher trainings… because mental health is not an easy thing though you can look at it and you think it is easy. It is not an easy thing” (Buwama Clinician, Baseline Interview).*

Training was also considered critical in the area of depression medications. In this case clinicians were concerned about their current inability to provide the best advice given their limited knowledge about the available drugs, particularly their side effects, their potential interactions with ART and their toxicity. One clinician highlighted some of these concerns:

*“… of course as you know about the tricyclic antidepressants they have [a] side effect–they can cause death; they are fatal if given in overdoses [and] for the [HIV] clients there is also taking Nevirapine and antidepressants. Now for Nevirapine, it reduces the concentration so you need to give a high dose of antidepressant” (Nkozi Clinician, Baseline Interview).*

The same clinician speaks to the current knowledge gaps among clinicians:

*“These drugs… do not have a clear literature about them. Though [for] fluoxetine they say it is safe in pregnancy they do not [provide] clear literature, so my challenge may come in giving antidepressants to pregnant mothers and lactating ones….”*

*Harmonisation in scheduling appointments.* In their narratives on changes needed at the organisational level, stakeholders–including patients and supervisors–essentially talked about the need for harmonisation in HIV and depression care. This, they proposed, would be achieved in different ways, including a departure from the current system in which the scheduling of clinician’s appointments assumes no correlation between HIV and depression. They argued that appointments for these two conditions need to be coordinated so that the patient can access treatment for both conditions on a single trip to the health facility.

*“It [clinician’s appointment for depression care] should actually depend on the period that you are given for the ART, say if you are given for three months, also the antidepressants should be given for three months such that the appointment dates are harmonized” (Nkozi, Antidepressants, Patient 1, Midline Interview).*

*“The biweekly [psychotherapy] appointments [for depression care] might be a challenge but I believe if we make it on a monthly basis so that if the patients come for ART treatment they even get depression treatment on the same day it will be easy for them to keep the appointment dates” (Focus Group Discussion with Lay Health Workers, Endline).*

*Structural changes.* Consistent with patients’ perspectives, some clinicians suggested structural changes in order to resolve the current disjointedness in service delivery, whereby HIV clinics are physically delinked from those for managing depression. These argued for more coordinated service delivery in the form of centralised physical spaces or what they referred to as one-stop centres, where patients would conveniently access both HIV and depression care.

*“I would prefer to have a one-stop centre for care. Like I am depressed, I need ART, [and] I get them from this dispensing window. Are you getting it? [But] now I would have to go to OPD to get the antidepressants because it can be dispensed in the OPD unlike the HIV drugs which cannot be dispensed anywhere…. I am suggesting that the dispensing log should also include the amitriptyline [antidepressants] here at the ART clinic so that when a client… comes here [s/he] does not need to go to another dispensing point” (Buwama Clinician, Midline Interview).*

### Patient level interventions to enhance knowledge of depression

Another necessary condition for successful integration of depression management into routine HIV care was to do with service users/patients. Stakeholders, particularly clinicians and patients, recognized that patients were sometimes not informed about depression as a health condition and needed to appreciate this reality in order for them to embrace the appropriate treatment. The stakeholders recommended enhancement of knowledge of depression among patients through educational and other awareness-raising interventions. They emphasized the need for health education targeted at the patients and the broader public through mass media, village sensitization meetings as well as patient follow-up to ensure understanding of depression and the related medication they were being asked to take.

*“I think informing the public through mass media such as televisions and radios can be of help…. Also holding sensitization meetings in the villages can be of great help” (Buwama, Antidepressants, Patient 2, Midline Interview).*

*“… we have to strengthen our follow up strategies that is the only way. Health education packaging… you know [so the] client understands why he takes the medicine and for how long. Then you make sure you make constant follow-up. You ensure follow up, they will be ok…” (Buwama Clinician, Baseline Interview).*

Aside from the organisational and patient level interventions that are necessary for success of integration, our formative research yielded some pertinent findings that pointed to necessary changes in the mode of delivery of depression management. These changes, which have now been incorporated in the HIV + D trial, relate to the decision support algorithm for the stepped care HIV + D intervention. While implementing the three-step intervention during the formative research, we observed that majority of participants who proceeded to Step II of the algorithm (57 out of 66 patients) preferred Behavioural Activation (psychotherapy) to antidepressant medication. For the purpose of the HIV + D trial, we have designed a more client-friendly decision support algorithm that provides for delivery of psychotherapy at Step II to all the participants who do not improve at Step I. This is instead of the earlier algorithm which gave patients the choice between psychotherapy and antidepressants at Step II. Consequently, antidepressant medication will now be provided together with psychotherapy at a higher level (Step III) for those patients whose depression scores remain high. Under the current algorithm, the stepped care intervention has four levels with referral to a mental health specialist being done at Step IV. We consider this adjustment in the decision support algorithm a necessary step in enhancing the feasibility of integrating depression into HIV care.

## Discussion

In this paper we explored stakeholders’ perspectives on the acceptability and feasibility of integrating the management of depression into routine HIV care in Uganda, drawing on data from PLWHA, clinicians, supervisors, lay health workers, and a sub-group of PLWHA, that is, adherent and non-adherent patients. Our findings demonstrate that integration, modelled along a realist approach that allows for contextual specificity and targets particular services, is acceptable among the stakeholders. This was more evidently so among those stakeholders who held the most direct stake in integration (service users [PLWHA] and clinicians), who credited the pilot intervention for improving ART adherence, overcoming sleeplessness and suicidal ideation, and regaining a sense of self-efficacy. These findings lend further credence to broader calls for integration of mental healthcare into primary healthcare as the strategy for addressing the significant unmet need for effective mental health services in low and middle income countries [35].

In the sub-Saharan African context where research on integrating services is still limited [36], the findings from this study contribute in strengthening the current evidence base to inform best practices in integrating depression management in HIV care. We have shown that stakeholders’ positive attitude towards integration was driven by a number of associated benefits, key among which was that the management of depression in the context of HIV care resulted in enhanced adherence to ART. Similar findings have been reflected in previous studies. In a 2017 cluster randomised controlled trial conducted in Uganda by Wagner and colleagues [37], depression treatment was associated with not only improved ART adherence but also retention in HIV care. Likewise, in a 2014 meta-analysis by Sin and DiMatteo [38] examining the same association, it was found that the odds of ART adherence were 83% better if the HIV positive individual received depression treatment.

But also worth noting is that while the association between depression treatment and ART adherence has been noted in previous studies, our study builds further on this evidence base by characterising some of the pathways through which such treatment leads to adherence. These notably included enabling disclosure in discordant relationships, which created an environment in which the HIV positive spouse was no longer encumbered by the need for secrecy in administering ART, and was also able to leverage the support of the spouse, for example in the form of reminders, to ensure they do not miss their dose. We view this as an important contribution insofar as facilitating better programming for HIV counselling to minimise the incidence of depression among PLWHA.

We also highlighted self-efficacy as one of the benefits accruing from depression treatment. Stakeholders, particularly PLWHA, credited the treatment for having enabled them to regain control over their lives by acquiring knowledge about a hitherto unknown disease (depression) they were suffering from, and by believing in their own agency in addressing their health problems. This finding echoes previous work as reported by Wagner et al. [37] in identifying the positive association between self-efficacy and ART adherence. But while Wagner et al. [37] point to the mediating role of self-efficacy, suggesting no effect of depression treatment on self-efficacy, we show in this paper that self-efficacy itself can be one of the benefits of depression treatment.

But our findings notably also highlight stakeholders’ view that while acceptable, the feasibility of integrating the management of depression into routine HIV care would necessarily be predicated on some key interventions. Guided by Benzer and colleagues’ [30] theoretical perspectives on factors influencing success of integrated care, we were able to identify organizational factors (namely training to develop clinical expertise), provider experiences (such as time pressures arising from redistribution of workloads) and patient factors (improving depression awareness) as some of the key areas that needed to be addressed as a precondition for the success of integration. These findings, particularly on the need for training, build further on those from earlier studies that have cited absence of depression care and expertise in HIV care programs [9, 36, 39, 40], and highlighted the need for HIV care providers to develop capacity to diagnose and manage depression [36]. Consistent with these findings, studies in Tanzania and Cameroon have shown that training HIV care providers in prescription and management of antidepressants is effective at improving depression outcomes [41, 42].

Stakeholders also echoed findings from earlier work [43] by advocating change in the form of raising patient (and the broader community) awareness about depression as a way of improving understanding of the disease and uptake of relevant services in the context of HIV care. This finding lends further credence to Benzer and colleagues’ [30] theoretical perspectives on the role of patient factors (in this case, knowledge about depression) or what they conceptualise as the surrounding environment in shaping success of integration.

Furthermore, the overwhelmingly positive attitude that clinicians expressed towards integration was notable in light of their concerns about the increased workload. We consider this attitude significant inasmuch as it raises the prospect of uptake of the innovation among clinicians whose support is critically important for the success of the innovation.

In terms of informing the HIV + D trial, our formative work has informed the design of a more client-friendly decision support algorithm that provides for four steps rather than the three that were delivered during the formative research. The current algorithm provides for screening and psychoeducation at Step I; delivery of psychotherapy at Step II to all participants whose depression scores remain high; delivery of both psychotherapy and antidepressants at Step III to those who scores are still high after Step II; and a referral to a mental health specialist at Step IV for those whose scores are persistently high after Step III.

## Limitations of the study

Our study had some limitations. One of these is that data were collected from only four HIV clinics in one district in central Uganda. While we make inferences from this study that may be generalizable to HIV care in Uganda–especially given the commonalities across the broader public health care system in the country, including the profile of the clientele normally attending these public facilities–we caution that these results may not exhaustively depict stakeholders experiences and perspectives across the country.

Another limitation of the formative study on which this paper is based is that the data collected on feasibility of the HIV + D integration model was qualitative and therefore did not include the economic aspect – the cost-effectiveness analysis. In this formative study, our focus was on technical and operational feasibility. Formative cost effectiveness studies including the local validation of health economics study tools were later undertaken and will be reported in a separate paper.

## Conclusion

Integrating the management of depression into routine HIV care in Uganda is acceptable among key stakeholders (patients and clinicians), but the feasibility of this innovation would require some changes at the organisational and patient levels. We have shown in the paper that stakeholders, most importantly service users (patients), attribute a number of benefits to integration, including improvement of ART adherence, overcoming sleeplessness and suicidal ideation, and regaining a sense of self-efficacy. We have also highlighted stakeholders’ concerns about the various challenges to integration, broadly categorised as organisational and patient-level factors, as well as their recommendations on how best to integrate. One key observation from our findings was that stakeholders who spoke most to the acceptability of integration were patients and clinicians. Acceptability among the patients was evident in the wide array of benefits they attributed to the HIV + D intervention in which they had a chance to experience integrated (depression and HIV) care. Similarly, we have noted that some clinicians demonstrated acceptability by supporting integration in spite of the extra workload it engendered. We consider the perspectives of these stakeholders an important indication of future uptake of integration considering that they are the most directly impacted and their cooperation arguably is critical for the success of integration. Given the paucity of research on service integration in sub-Sahara Africa [36] our findings will feed into the much needed knowledge base for the development of evidence-based and contextually grounded models of integration.

## Data Availability

The datasets used and/or analysed during the current study are available from the corresponding author on reasonable request.

## Abbreviations

HIV/AIDS: Human immune deficiency virus/Acquired immune deficiency syndrome or acquired immunodeficiency syndrome
ART: Antiretroviral therapy
PLWHA: Persons living with HIV and AIDS
STD: Sexually transmitted disease
HIV + D: Integrating the management of depression into routine HIV care in Uganda
